# Social neuroscience: the second phase

**DOI:** 10.3389/fnhum.2013.00020

**Published:** 2013-02-06

**Authors:** Chad E. Forbes, Jordan Grafman

**Affiliations:** ^1^Department of Psychology, University of DelawareNewark, DE, USA; ^2^Rehabilitation Institute of ChicagoChicago, IL, USA

The systematic examination of how social psychological phenomena can be informed by neuroscience methodologies, and how our understanding of neural function can be informed by social psychological research, began approximately 20 years ago. Increased interest in these topics largely coincided with methodological advances in electroencephalography (EEG) and functional magnetic resonance imaging (fMRI). The desire to understand how and where the world around us is represented in the brain (and vice versa) ultimately spawned a field of research that is thriving today: social neuroscience.

Many early social neuroscience studies were concerned with the basic question of where social-oriented phenomena are represented in the brain. While these studies were obviously of paramount importance for the development of the field, more recently this approach has been likened to modern day phrenology by critics. It is important to note, however, that these early studies were necessarily constrained by methodological and analytical approaches of the time. As these methodological and analytical approaches have evolved, so too has the field of social neuroscience. The ability to assess how different neural regions interact on the order of milliseconds has been particularly important for enhancing our understanding of both the complexity of the brain and the complexity inherent in any given social interaction or social perceptual process.

This second phase of social neuroscience, which focuses less on where things are happening in the brain and more on how regions of the brain form networks that interact to engender a psychological process, is poised to have a big impact on existing theories in social psychology. The notion that many different neural regions necessarily interact almost instantaneously and continuously throughout a given cognitive process seems perfectly sensible from a neuroanatomical perspective, but the ramifications this perspective has for prevailing theories in social psychology are pronounced. For instance, take the dual process perspective, i.e., the theory that many, if not all, social cognitive processes (e.g., attitudes, prejudice, attributions, etc.,) are uniquely influenced by implicit\automatic\fast\subconscious processes that occur outside of an individuals' conscious awareness and are uncontrollable, and explicit\controlled\slow\conscious processes that an individual has conscious access to and can control. According to the dual process account, implicit and explicit processes are orthogonal to one another.

From a social neuroscience perspective this would suggest some kind of neuroanatomical distinction between implicit and explicit processes as well. Indeed, there is evidence to suggest some functional and neural specificity with regards to implicit and explicit processes. For example, the amygdala has been linked to many implicit social processes such as automatic stereotype activation and perceptions of facial trustworthiness (Cunningham et al., 2004; Todorov and Engell, 2008; Forbes et al., 2012a,b). The orbitofrontal cortex (OFC) appears to be integral for regulating implicit processes and fear conditioned responses, particularly those associated with visceral arousal stemming from the medial temporal lobe, within the context of current goal states (e.g., regulating negative stereotype activation or extinguishing learned fear responses, Soliman et al., 2010; Forbes et al., 2012a,b). As the OFC is highly interconnected with regions in the medial temporal lobe like the amygdala and lateral prefrontal cortical regions such as dorsolateral prefrontal cortex (DLPFC), the functional specificity of the OFC with regards to implicit processing again seems perfectly logical (Rolls and Grabenhorst, 2008). Likewise, as DLPFC is considered a hub for executive function and conscious control of behavior and thoughts, the DLPFC must play an integral role in explicit processes in general such as the generation of explicit attitudes and beliefs, and conscious perceptions of others. Indeed, a bevy of social neuroscience studies implicate this region in explicit social cognitive processes specifically (e.g., Richeson et al., 2003; Cunningham et al., 2004; Forbes and Grafman, 2010; Forbes et al., 2012a,b).

Where the waters become much murkier so-to-speak, is when one considers the time at which these processes unfold. A fundamental assumption of dual-process theories is that time is one of *the* critical determinants of whether social cognition is influenced by implicit or explicit processes. Whereas implicit processes (and products of these processes) occur when individuals make perceptions or decisions quickly, explicit processes can only manifest when one has ample time. This assumption, however, is not consistent with known anatomical and neural conductive properties, where functionally distinct regions of the brain are highly interconnected with one another and neural propagation of action potentials can occur on the order of 0.5–50 ms within the cortex (Fuster, 1997; Buzsaki, 2006). How then can we disentangle the undoubtedly complex relationships inherent in the psychological interplay between implicit and explicit processes? Possibly via assessing interactions between neural correlates that represent these psychological processes (e.g., assessing how the temporoparietal junction and medial PFC, two regions thought to be integral for theory of mind, interact to influence theory of mind processes).

## Assessing interactions between neural regions

**Figure 1:**
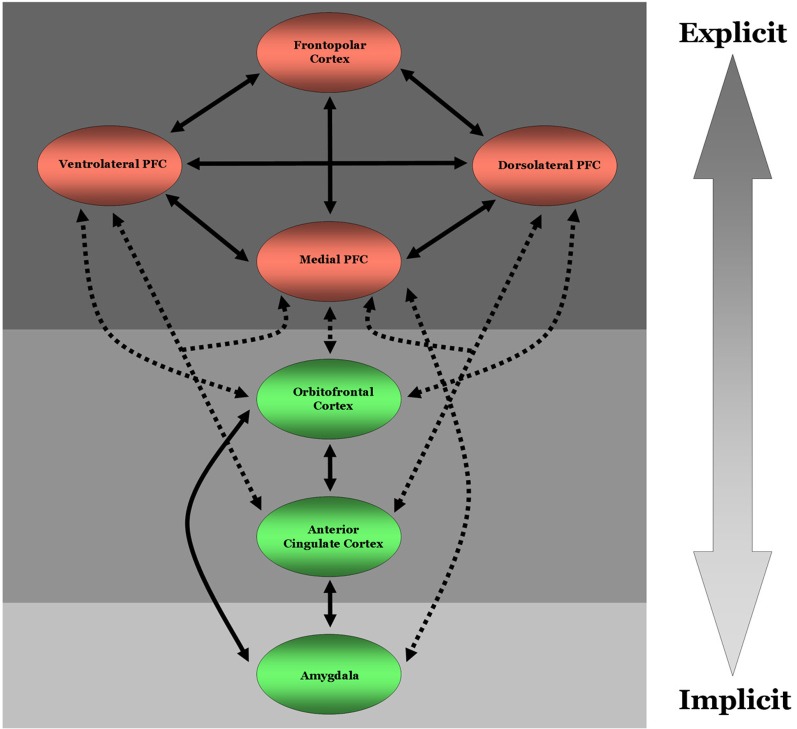
**Diagram of direct neural connectivity between regions critical for implicit and explicit social cognitive and moral judgment processes.** Solid bi-directional arrows denote direct reciprocal neural connectivity between two regions within a given processing system, i.e., implicit or explicit processing. Dashed bi-directional arrows represent direct reciprocal connectivity between two regions typically involved in either implicit or explicit processing. Green colored circles denote neural regions that are typically involved in more implicit cognitive processing. Red colored circles signify neural regions that are typically involved in explicit cognitive processing. These regions are not exclusively involved in implicit or explicit processing, however. This conjecture is represented by the three shaded boxes and large arrow on the right. The lightest gray box represents neural regions, namely the amygdala here for the sake of simplicity, that are largely involved in implicit processes. Likewise, the darkest gray box highlights neural regions largely involved in explicit processes. The medium shaded box represents regions that have been shown to be recruited during both implicit and explicit processing. PFC, prefrontal cortex.

Gaining a better understanding of how neural regions integral for implicit or fast cognitive processing (e.g., the amygdala or anterior cingulate cortex), interact with regions integral for explicit or slower cognitive processing (e.g., lateral PFC regions; although note that PFC activations likely precede many routine/well-rehearsed daily life activities suggesting the timing of PFC activation is context dependent) early and often during the social informational processing stream can provide valuable insight in to the extent to which any specific social process is influenced by implicit and explicit social cognitive processes (Figure 1). One way this can be achieved is by examining the degree to which collections of neurons in different regions of cortex fire at a specific rate (i.e., frequency) and in synchrony with one another, i.e., by performing coherence analyses. A growing body of evidence indicates that coherence between two neural regions reflects the degree to which they are communicating with one another (Engel and Singer, 2001; Buzsaki, 2006; Siegel et al., 2011, 2012). This communication, in turn, has been associated with more efficacious cognitive processing, e.g., working memory, encoding, and error detection (Cavanagh et al., 2009; Benchenane et al., 2011), and top–down modulation of visual and working memory networks by prefrontal cortex (Zanto et al., 2011). While it is important to stress that coherence between regions depends at least in part on sustained networking as opposed to transient bindings, the effects of enhanced coherence between distant regions on behavior can occur almost instantaneously and throughout the information processing stream. Such observations directly contradict what one would expect if implicit and explicit processes are orthogonal to one another.

Findings from the fMRI literature, which utilizes ever-evolving analytic strategies to examine the functional connectivity between different neural regions (He et al., 2011), are also adding to our understanding of the complexity of the social brain. These analytic strategies are changing our perceptions of the functions of the brain from one where distinct neural regions perform uniform tasks independent of context to one that emphasizes the dynamic properties of each region that differentially interact with one another as a function of context. For instance, individuals with egalitarian motivations can successfully down-regulate amygdala activation in response to novel black faces via increased dorsolateral PFC activity in stereotype neutral contexts (Cunningham et al., 2004; Forbes et al., 2012a). When negative black stereotypes are primed specifically, however, i.e., the context in which the faces are presented has been altered such that novel black faces might be perceived in a negative stereotypic manner, amygdala activation is not down-regulated, even when individuals are given more time to process the faces. Instead, amygdala activation persists in to explicit processing speeds and initiates a dynamic interaction between the OFC and DLPFC that ultimately engenders stereotype-consistent perceptions of novel black faces (i.e., participants report seeing more angry black faces compared to white faces, even though all faces had neutral expressions; Forbes et al., 2012a). Importantly, the interaction between these regions normally thought to be uniquely involved in implicit and explicit processing occurs regardless of whether faces are presented subliminally or supraliminally; a finding that again speaks against the argument that implicit and explicit social cognitive processes are orthogonal to one another.

### How genetic polymorphisms may alter neural interactions

Further complicating our understanding of how different neural regions interact with one another to shape our perceptions of the social world, but immensely enriching it nonetheless, are recent advances in the burgeoning field of genetics. While far less understood, it is becoming clear that genetic polymorphisms in genes integral for neural function moderate the interaction between and within different neural regions involved in the processing of social information. Perhaps one of the more provocative examples of this stems from findings indicating that different polymorphisms in the brain derived neurotrophic factor (BDNF) gene, a gene that promotes neuroplasticity throughout the brain, have been associated with greater connectivity between and within different neural regions. For instance, different BDNF polymorphisms have been associated with increased connectivity between the amygdala and VMPFC and subsequently individuals' ability to extinguish learned fear responses (Soliman et al., 2010). BDNF induced plasticity within neural regions such as OFC and DLPFC have also been found to moderate individuals' ability to inhibit implicit and explicit bias respectively (Forbes et al., 2012b).

Given the seemingly ubiquitous role of the amygdala, PFC and amygdala-PFC connectivity in social cognition (e.g., stereotype activation and regulation, emotional expression and regulation, perceptions of trustworthiness, attribution, attitudes, etc.), BDNF-induced variation in plasticity may play an important role in explaining individual differences between and within groups on a variety of social psychological dimensions ranging from prejudice to political orientation to moral judgment. One critical area for future research is determining how exactly BDNF is modulating social cognitive processes. There are three particularly important questions that should be addressed. (1) Do different BDNF polymorphisms alter the plasticity between representations of a given construct like those between attributes associated with a given ethnic group (i.e., actually strengthen stereotypic associations)? (2) Do they alter plasticity within and between regions that are necessary to regulate cognitive processes? Or (3) Do they influence both equally or differentially? Findings from Forbes et al. (2012b) provide evidence supporting the second question, but much more research will be necessary to fully understand how BDNF polymorphisms alter associative and regulatory strength of social representations.

BDNF is not the only gene known to influence connectivity between the amygdala and other neural regions, however. In addition to the effects of BDNF on amygdala-PFC connectivity, polymorphisms in the catechol-O-methyltransferase (COMT) and serotonin transporter gene have been shown to modulate affective arousal and regulation as well as the retention of fear extinguished memories (Drabant et al., 2006; Hartley et al., 2012). While primarily studied within the context of affective disorders, it is clear to see how these findings also have important implications for future work in social neuroscience. Given that many social interactions are likely to initiate a cascade of implicit and explicit processes that would invariably rely on amygdala-PFC interactions, it stands to reason that polymorphisms in genes such as COMT, serotonin and BDNF could have subtle influences on behavior in a given situation. For example, both blacks and whites have been shown to establish learned fear responses to novel members of their ethnic outgroup faster, and have greater difficulty extinguishing these learned fear responses (Olsson et al., 2005). Findings from the literature described above would suggest then that some people would be either better or worse at extinguishing the learned outgroup fear responses based on polymorphisms in COMT, serotonin and/or BDNF; a conjecture that has direct implications for intergroup relations, prejudice and prejudice reduction strategies.

These findings, and the current state of the field for that matter, represent the tip of the iceberg with many fruitful avenues for future research. For example, current as well as future research (including a study in this research topic: See Krueger et al., 2012) is examining the role of polymorphisms in the oxytocin receptor gene in facilitating trust and social bonds. This research will likely shed light on individual differences in trustworthiness and attachment, i.e., the foundations of human society. It is equally likely that myriad discoveries of other genetic polymorphisms are imminent and will undoubtedly impact the field of social neuroscience in substantial ways.

Thus, the field of social neuroscience stands to benefit greatly from analytical and theoretical advances in neuroscience and cognitive neuroscience and should utilize the analytic strategies mentioned above to inform theories integral to the field of social psychology. In this vein, the field of social neuroscience has the potential to make dramatic contributions to social psychological theory. Articles in this research topic employ these methods and provide further evidence across a variety of domains in social psychological research that supports this conjecture.

Using EEG methodologies to further blur the lines between implicit and explicit processes, Forbes et al. (2012c) discuss EEG and lesion studies that find surprising overlap and coherence between neural regions on tasks thought to uniquely recruit implicit and explicit processes (the IAT). Similarly, Cunningham et al. (2012) highlight the dynamic, context-dependent modulation of neural processes involved in social perception in an EEG study that finds that motivational orientation alters the rapid processing of ethnic ingroup and outgroup faces such that white and black faces are perceived similarly when white individuals are motivated to approach black faces. Providing further evidence for how the interaction between implicit and explicit processes may modulate person perception, Poore et al. (2012) report an fMRI study that examines how implicit reward processing in the striatum predicts decreases in explicit trust toward close others when individuals received information from these sources that violated their expectations. Harada et al. (2012) also use fMRI to demonstrate how situational (perceived power) and sustained social factors (cultural stereotypes) interact to modulate regions integral for both math calculation and implicit and explicit processes. Beasley et al. (2012) and Hecht et al. (2012) add breadth to our understanding of the complexity of brain by situation interactions utilizing an evolutionary approach.

Studies presented in this research topic also provide new insight in to our understanding of the role genetic polymorphisms play in social cognition. Richter et al. (2011) demonstrate that a polymorphism in AKAP5 is associated with both explicit reports of aggressive behavior, anger expression and anger control, and implicit regulation of anger. These differences manifested at the neural level as well, implicating enhanced activation in ACC during the processing of angry faces among individuals with the polymorphism associated with decreased aggression. Specific to trust and the facilitation of social bonds, Krueger et al. (2012) report a study that identifies different polymorphisms in the oxytocin receptor gene associated with trusting behaviors specifically. While these findings undoubtedly represent the gateway to understanding highly complex gene-environment interactions, e.g., environmental exposures can also modulate the instructions that go from the gene to the neuron and related cells and essentially override a predisposition using epigenetic means (Rutter et al., 2006), both studies nicely exhibit how genetic polymorphisms can nonetheless affect social behavior in meaningful ways. Consistent with this, Falk et al. (2012) provide a critical examination and organizing framework for understanding how genetic polymorphisms that moderate neurochemical responses in the brain may interact with known neural networks to predispose individuals to social influences and conformity. Ratner and Kubota (2012) also highlight the promise of genetic contributions specific to the study of intergroup relations but eloquently, and rightfully, stress caution in these approaches as well.

The research and reviews presented in this research topic represent the second phase of social neuroscience. That is, they focus less on where things are happening in the brain and more on how different neural regions interact as a function of context and genetic predispositions almost instantaneously in a given situation to modulate social perceptual processes and behavior. While these forays will likely engender an appreciation of the mind-numbing complexity of dynamic gene-neural-situational interactions and their behavioral byproducts, the current steps being made toward this progress are obviously imperative. As such, this is an exciting time for social neuroscience as a field as the products of these endeavors will no doubt have a dramatic impact on theories integral for social and cognitive psychology and neuroscience for years to come.
